# Impact of invasive bladder cancer and orthotopic urinary diversion on general health-related quality of life: An SF-36 survey

**DOI:** 10.3892/mco.2013.125

**Published:** 2013-05-20

**Authors:** MINGYING YANG, HAIFENG WANG, JIANSONG WANG, MINGHUI RUAN

**Affiliations:** 1 Departments of Nursing, The Second Affiliated Hospital of Kunming Medical University, Yunnan Institute of Urology, Kunming, Yunnan 650101, P.R. China; 2 Urology, The Second Affiliated Hospital of Kunming Medical University, Yunnan Institute of Urology, Kunming, Yunnan 650101, P.R. China

**Keywords:** bladder neoplasms, orthotopic, urinary diversion, quality of life, SF-36 general health survey

## Abstract

Bladder cancer is a common type of genitourinary cancer, and radical cystectomy with urinary diversion is considered to be the most effective local treatment for invasive bladder cancer. In order to assess the functional results and health-related quality of life (QOL) in bladder cancer patients with an orthotopic neobladder, and to provide a reasonable basis for the evaluation of urinary diversion *in situ*, we conducted a study on 96 neobladder patients. In December, 2011, questionnaires were mailed to 96 patients that had undergone urinary diversion surgery between January, 2007 and December, 2009. The questionnaire included the validated health-related QOL questionnaire and the MOS 36-item Short-Form Health Survey (SF-36). We compared the functional results between patients with an orthotopic neobladder and those with other types of urinary diversion at 6, 12 and 24 months after surgery. Data from 82 patients (54 with orthotopic and 28 with non-orthotopic urinary diversion) were included in the analysis. The SF-36 patient scores following orthotopic urinary diversion were significantly higher than those following non-orthotopic urinary diversion. The majority of patients with orthotopic urinary diversion considered themselves as healthy and their scores of total health were higher compared to those of patients with non-orthotopic urinary diversion. However, there were no differences in the scores of physical functioning between patients with orthotopic and those with non-orthotopic urinary diversion. Our findings regarding health-related QOL and the frequency of complications in the orthotopic and the non-orthotopic urinary diversion groups were similar. However, the mental health of patients with orthotopic urinary diversion was more easily restored compared to that of patients with non-orthotopic urinary diversion, which reduced their overall recovery time.

## Introduction

Bladder cancer is a common type of genitourinary cancer. Radical cystectomy with urinary diversion is considered to be the most effective local treatment for invasive bladder cancer (1,2). As regards quality of life (QOL), including urinary, sexual and social functioning, daily living activities and body image satisfaction, orthotopic continent diversions are considered the ‘gold standard’ among reconstructive procedures (3–5). However, QOL has not been sufficiently assessed in non-metastatic bladder cancer patients (NMBC) (6). Therefore, available data on the comparison of the impact of different treatments on the QOL of NMBC patients are limited (7). The MOS 36-item Short-Form Health Survey (SF-36) score is extensively used worldwide and is considered to be accurate (8–11). SF-36 has become a standard questionnaire and it may be applied to patients as well as to healthy subjects. In addition, the questions may be easily answered within a short time and they are limited to the evaluation of basic issues regarding overall health. In this study, we compared the functional results between patients with an orthotopic and those with a non-orthotopic neobladder at 6, 12 and 24 months after surgery, using the SF-36 method.

## Patients and methods

### Patients

Between January, 2007 and December, 2009, an orthotopic neobladder reconstruction was performed on 54 patients (experimental group). In addition, non-orthotopic neobladder surgery (cutaneous diversion), was performed in 28 patients (control group). The experimental group included 50 patients with transitional cell carcinoma (5 with T1, 31 with T2 and 14 with T3), 2 with squamous cell carcinoma, 1 with adenocarcinoma and 1 with neuroendocrine carcinoma (mean age of the 54 patients, 66.0 years). The control group included a total of 28 patients with a non-orthotopic neobladder, 27 of which had transitional cell carcinoma (14 had T2, 12 had T3 and 1 had T4) and 1 had adenocarcinoma (T2) (mean age of the 28 patients, 65.6 years). The 82 patient samples were all confirmed by pathological examination following surgery. This study complies with current ethical considerations. The protocol was approved by the Ethics Committee of Kunming Medical University. Informed consent was obtained from all patients.

### Methods

The functional results of the two groups were compared at 6 months, 1 year and 2 years after surgery, using the SF-36 survey method. Regarding the orthotopic reconstruction, most of the surgical techniques were based on Studer’s method. At a time point of >6 months after surgery, the SF-36 survey was conducted by mail to determine the QOL of patients with an orthotopic and that of patients with a non-orthotopic neobladder. The SF-36 survey consisted of 36 questions and the 2nd question was a self-evaluation of health status, which was not included in the score calculation. We assessed 8 aspects of health-related QOL: physical functioning, role-physical functioning, bodily pain, general health, vitality, social functioning, role-emotional functioning and mental health. The number of questions regarding each health concept ranged from 2 for social functioning and bodily pain to 10 for physical functioning and the number of response options per question ranged from 2 (yes or no) to 6 (none, very mild, mild, moderate, severe or very severe). The score per question was estimated for each health concept, ranging from 0 to 100, with higher scores indicative of an improved outcome. For the orthotopic neobladder patients, a detailed continence questionnaire (not validated) including voiding questions was examined on the same day as the SF-36 survey.

### Statistical analysis

Descriptive data were reported as the means ± SD. The differences in the mean values between the ileal neobladder and the cutaneous diversion patients were analyzed by the Mann-Whitney U test. Data regarding the percentage of patients with a neobladder, the number of males, the number of patients with a pathological stage T3 or higher and the current disease status were analysed by the χ^2^ test. P<0.05 was considered to indicate a statistically significant difference.

## Results

### Comparison of data from the SF-36 survey between the experimental and control groups at the time point of 6 months

**Figure 1.**
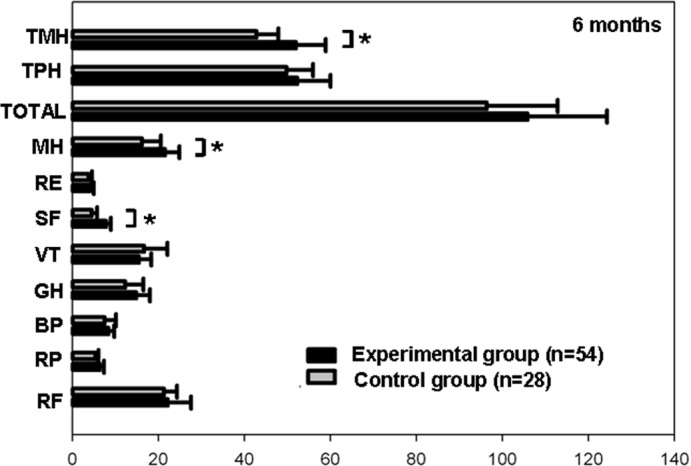
Comparison of data from the Short-Form Health Survey (SF-36) between the experimental and control groups at 6 months (*P<0.05). TMH, total mental health; TPH, total physical health; MH, mental health; RE, role-emotional functioning; SF, social functioning; VT, vitality; GH, general health; BP, bodily pain; RP, role-physical functioning; RF, physical functioning.

All 82 patients (100%) were available for assessment at the time point of 6 months. A total of 51 out of the 54 patients in the experimental group (94.4%) and 26 out of the 28 in the control group (92.9%) were available for assessment at the time point of 12 months. A total of 45 patients in the experimental group (83.3%) and 23 in the control group (82.1%) were available for assessment at the time point of 24 months. All questionnaires were qualified. At the time point of 6 months, the response rate was similar for the experimental group (54/54, 100%) and for the control group (28/28, 100%). The majority of patients (76/82, 92.7%) indicated that their physical condition was worse compared to 1 year before when were the questionnaires completed. Data from the SF-36 survey are provided in Table I and Fig. 1.

### Comparison of data from the SF-36 survey between the experimental and control groups at the time point of 12 months

**Figure 2.**
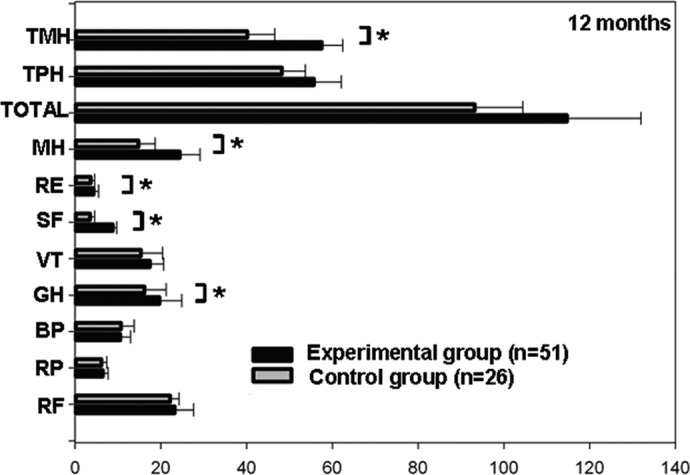
Comparison of data from the Short Form Health Survey (SF-36) between the experimental control groups at 12 months (*P<0.05). TMH, total mental health; TPH, total physical health; MH, mental health; RE, role-emotional functioning; SF, social functioning; VT, vitality; GH, general health; BP, bodily pain; RP, role-physical functioning; RF, physical functioning.

The social functioning and mental health scores in the experimental group were significantly higher compared to those in the control group (P<0.05) and the QOL score in the experimental group was higher compared to the control group; however, the difference was not statistically significant (P>0.05). At the time point of 12 months, the response rates of the experimental group and the control group were 51/54 (94.4%) and 26/28 (92.9%), respectively. In the experimental group, 1 patient succumbed to multiple metastases of bladder cancer, 1 expired due to non-cancerous causes and 1 was lost to follow-up. In the experimental group, 22 patients (43.1%) indicated that their physical condition was worse compared to 1 year before, 27 (52.9%) indicated that their physical condition had improved compared to 1 year before and 2 (3.9%) observed no differences compared to 1 year before when they completed the questionnaires. In the control group, 16 patients (61.5%) indicated that their physical condition was worse compared to 1 year before and 10 (38.5%) indicated that their physical condition had improved compared to 1 year before when they completed the questionnaires. Data from the SF-36 survey are provided in Table II and Fig. 2. The general health, social functioning, role-emotional functioning and mental health scores in the experimental group were significantly higher compared to those in the control group (P<0.05). The QOL score in the experimental group was higher compared to that in the control group; however, the difference was not statistically significant (P>0.05).

### Comparison of data from the SF-36 survey between the experimental and control groups at the time point of 24 months

**Figure 3.**
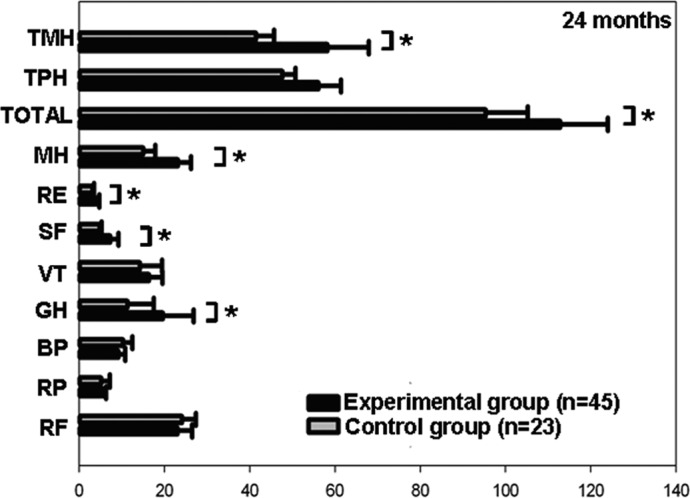
Comparison of data from the Short Form Health Survey (SF-36) between the experimental control groups at 24 months (*P<0.05). TMH, total mental health; TPH, total physical health; MH, mental health; RE, role-emotional functioning; SF, social functioning; VT, vitality; GH, general health; BP, bodily pain; RP, role-physical functioning; RF, physical functioning.

At the time point of 24 months, the response rates of the experimental and control groups were 45/54 (83.3%) and 23/28 (82.1%), respectively. In the experimental group, one patient succumbed to multiple metastases of bladder cancer and 5 patients died due to non-cancerous causes. In the control group, 2 patients succumbed to non-cancerous causes and 1 was lost to follow-up. In the experimental group, 8 patients (17.8%) indicated that their physical condition was worse compared to 1 year before, 11 (24.4%) indicated that their physical condition had improved compared to 1 year before and 26 (57.8%) observed no differences compared to 1 year before when they completed the questionnaires. In the control group, 14 patients (60.9%) indicated that their physical condition was worse compared to 1 year before, 7 (30.4%) indicated that their physical condition had improved compared to 1 year before and 2 (8.7%) observed no differences compared to 1 year before when they completed the questionnaires. Data from the SF-36 survey are provided in Table III and Fig. 3. The general health, social functioning, role-emotional functioning, mental health and QOL scores in the experimental group were significantly higher compared to those in the control group (P<0.05).

## Discussion

Urinary reconstruction is indispensable following radical cystectomy for invasive bladder cancer (5,12). In our institute (Yunnan Institute of Urology, China), orthotopic neobladder reconstruction procedures were performed when the patients had the appropriate indications for this type of surgery from the viewpoint of cancer control, as well as when the patients requested the surgery. However, we performed non-orthotopic neobladder procedures when the patients did not have the appropriate indications for the construction of an ileal neobladder. Due to the increasing number of patients receiving an orthotopic rather than a non-orthotopic neobladder following radical cystectomy, it is significant to accurately demonstrate the health-related QOL differences between the two procedures, in order to select the patient-appropriate type of urinary diversion, considering that they exhibit similar morbidity and mortality rates. Although QOL is increasingly recognized as an important outcome measure following treatment for urological malignancy, the QOL findings after a cystectomy remain a controversial subject.

The health-related QOL, as well as the micturition status and continence following a cystectomy, are important considerations in selecting the optimal treatment modality (13,14). Therefore, we investigated whether there were differences in QOL between the experimental and the control groups. Numerous questionnaires have been developed in order to evaluate the health-related QOL. The health-related QOL in patients with urinary reconstructions has been evaluated thus far using various types of questionnaires (15). The SF-36 score is extensively used worldwide and is considered to be accurate (16). Furthermore, it is a standard questionnaire that may be applied to patients as well as to healthy subjects. In addition, the questions may be easily answered within a short time and they are limited to the evaluation of basic issues regarding overall health.

Our data demonstrated that more patients perceived themselves as healthy following an orthotopic urinary diversion and their total health scores were higher compared to those of patients undergoing a non-orthotopic urinary diversion; however, there were no differences in the scores of physical functioning between the two groups. There were no statistical differences in the health-related QOL between the orthotopic and the non-orthotopic neobladder group; however, the functional results of orthotopic neobladder patients were satisfactory and consistent with those reported by other studies. In conclusion, the orthotopic neobladder reconstructive surgery may be performed on patients with the appropriate indications from the viewpoint of cancer control, particularly on patients concerned over a negative body image due to the presence of a urinary stoma. Our data may assist patients in the selection of the most appropriate treatment option. However, studies including larger patient samples may help elucidate the relative psychological benefits associated with various types of urinary tract reconstruction.

## Figures and Tables

**Table I.** Comparison of data from the Short Form Health Survey (SF-36) between the experimental and control groups at 6 months.

Items	Experimental group (n=54)	Control group (n=28)	P-value
Physical functioning	22.13±5.42	21.26±3.04	0.27
Role-physical functioning	6.32±1.04	5.34±0.74	0.08
Bodily pain	8.36±1.41	7.52±2.63	0.29
General health	14.82±3.19	12.26±4.27	0.14
Vitality	15.49±2.88	16.62±5.43	0.31
Social functioning	7.83±1.15	4.49±1.29	0.01
Role-emotional functioning	3.96±1.03	3.73±0.82	0.77
Mental health	21.49±3.37	16.20±4.33	0.02
Overall health	105.80±18.49	96.36±16.42	0.11
Total physical health	52.23±7.74	49.76±6.20	0.32
Total mental health	51.93±6.91	42.77±5.12	0.01

**Table II.** Comparison of data from the Short Form Health Survey (SF-36) between the experimental and control groups at 12 months.

Items	Experimental group (n=51)	Control group (n=26)	P-value
Physical functioning	23.22±4.37	22.14±2.03	0.17
Role-physical functioning	6.46±1.18	6.07±1.24	0.26
Bodily pain	10.47±2.43	10.64±3.06	0.63
General health	19.64±5.23	16.18±5.01	0.03
Vitality	17.38±3.27	15.33±5.06	0.11
Social functioning	8.72±0.96	3.47±1.04	0.01
Role-emotional functioning	4.29±1.17	3.62±0.93	0.01
Mental health	24.36±4.74	14.71±3.89	0.01
Overall health	114.7±17.32	93.17±11.24	0.11
Total physical health	55.69±6.34	48.17±5.47	0.08
Total mental health	57.49±4.93	40.16±6.36	0.01

**Table III.** Comparison of data from the Short Form Health Survey (SF-36) between the experimental and control groups at 24 months.

Items	Experimental group (n=45)	Control group (n=23)	P-value
Physical functioning	23.07±3.44	24.15±3.28	0.43
Role-physical functioning	5.79±0.54	5.21±1.96	0.26
Bodily pain	9.27±1.54	10.33±2.17	0.56
General health	19.63±7.26	11.35±6.20	0.01
Vitality	16.37±3.19	14.28±5.17	0.06
Social functioning	7.32±1.94	4.86±0.46	0.01
Role-emotional functioning	3.92±0.81	3.12±0.42	0.01
Mental health	23.17±3.06	15.10±2.79	0.01
Overall health	112.8±11.23	95.41±9.86	0.01
Total physical health	56.13±5.31	47.67±3.12	0.09
Total mental health	58.22±9.71	41.47±4.29	0.01
